# Expression of the chemokine receptor CXCR4 in human hepatocellular carcinoma and its role in portal vein tumor thrombus

**DOI:** 10.1186/1756-9966-29-156

**Published:** 2010-11-27

**Authors:** Nan Li, Weixing Guo, Jie Shi, Jie Xue, Huasheng Hu, Dong Xie, Mengchao Wu, Shuqun Cheng

**Affiliations:** 1 Department of Oncological Comprehensive Treatment, Eastern Hepatobiliary Surgery Hospital, Second Military Medical University, Shanghai 200438, China; 2 Institute of Nutritional Sciences, Chinese Academy of Science, Shanghai 200032, China

## Abstract

**Background:**

This study was conducted to investigate the expression of CXCR4 in portal vein tumor thrombus (PVTT) tissue and its possible role in the invasiveness of tumor thrombus cells.

**Methods:**

We detected differential expression of CXCR4 between PVTT and hepatocellular carcinoma (HCC) by an immunohistochemical assay. Lentivirus-mediated RNA interference and a migration assay were performed on human primary cells derived from PVTT to study the impact of CXCR4 on the invasiveness of HCC.

**Results:**

The expression of CXCR4 in tumor thrombus tissue was higher than that in HCC tissue. The invasion ratio of PVTT cells was significantly decreased (P < 0.05) after being infected with a CXCR4-targeting siRNA lentivirus, indicating that downregulation of CXCR4 by lentivirus-mediated RNA interference significantly impaired the invasive potential of PVTT.

**Conclusions:**

These results indicate that CXCR4 is an effective curative target for hepatocellular carcinomas with PVTT.

## Background

Hepatocellular carcinoma (HCC) is an extremely common malignant tumor. China is developing one of the highest incidences of liver cancer worldwide. About 45% of de novo HCC cases were discovered in mainland China, and about 110, 000 people died of hepatoma. In fact, in Asian countries such as China, India, the Republic of Korea, Singapore and Vietnam, more than 80% to 90% cases of HCC were associated with human hepatitis B virus (HBV) infections. More than 2 billion people have been infected with HBV worldwide, and more than 350 million of these people became infectors; 75% of all infectors live in Asia, and 33% live in mainland China [1,2]. In fact, chronic HBV infection greatly increases the risk of liver cirrhosis and HCC, resulting in the deaths of nearly one million HBV infectors from a variety of liver diseases, such as hepatic failure, hepatic cirrhosis and HCC.

For the past several years, even though the therapy provided to HCC patients has greatly improved, most patients in the middle and advanced stages of HCC have generated portal vein metastases, which form portal vein tumor thrombi (PVTT)[3]. The resected sample ratio of clinical surgery for the liver is relatively low, and the recurrence ratio after surgery is relatively high [4]. Overall, the total curative effect in these cases is not as good as expected. The most common reason for carcinoma metastasis is that the cancer cells grow toward the portal vein, which leads to the formation of PVTT. Studies on the mechanisms of tumor formation and metastasis are hindered by difficulties with the corresponding HCC cell lines [5].

The CXCR4 protein is a G protein-coupled, seven-transmembrane receptor. The chemokine CXCL12, also called stromal-derived factor (SDF-1), is the sole ligand for CXCR4 [6]. Unlike other chemokines and their receptors, CXCR4 and SDF-1 are constitutively expressed in a variety of tissues, including the brain, heart, liver, lung, spleen and kidney [1,7,8]. SDF-1 is expressed in hematopoietic and non-hematopoietic tissues and was originally identified from bone marrow stromal cells as a pre-B cell growth factor, which is essential for heart, nervous system and blood vessel development. Mice with a targeted deletion of the CXCL12 gene die perinatally, whereas the CXCR4 protein is expressed mainly in neutrophilic granulocytes, macrophages and dendritic cells. The interaction between CXCR4 and SDF-1 plays an important role in the formation of embryos, the development of blood vessels and the heart, the homing of hematopoietic stem cells after transplant, the transmembrane migration of inflammatory cells, T lymphocyte proliferation and the inflammatory response. After further research on the receptor, investigators found that CXCR4 is one of the most comprehensive cytokine receptors expressed in tissue, playing an important role in the growth and metastasis of a variety of malignant tumors [9].

In this article, through *in vitro *primary culture methods, we obtained an HCC cell line derived from the human hepatoma portal vein, which provided the experimental materials for a functional study of the role of CXCR4 in tumor cell invasiveness. To confirm the novel role of CXCR4 in hepatocarcinogenesis, the expression levels of CXCR4 in tumor tissue, adjacent hepatic tissue and PVTT tissue were measured. Finally, the mutual effects of CXCR4 expression and clinical pathology characteristics were discussed [10]. To further investigate the role of CXCR4 in HCC tumorigenesis and metastasis, a migration assay was performed on PVTT cells following the suppression of CXCR4 expression by the lentivirus-mediated expression of small hairpin RNA (shRNA).

## Methods

### Patients

#### Patient sample exhibiting HCC with PVTT

A total of 23 cases originated from the resected sample of HCC of active hepatitis combined with PVTT in the Eastern Hepatobiliary Surgery Hospital from May 2007 to May 2008. Of all of the cases, 14 cases were male and 9 were female, and the ages ranged from 28 to 66 years, with an average age of 42. The detection of hepatitis B DNA in all patients was greater than 10^4 ^(10^4^-10^7^) copies/ml. Nineteen of the patients had HbsAg (+), HbeAg (+) and HbcAg (+), which accounted for 82.6% of the patients; 4 cases were HbsAg (+), HbeAb (+), HbcAg (+), which accounted for 17.4%. There were 7 cases with complicating lesser tubercle hepatic cirrhosis, 10 cases with tuberculum majus liver cirrhosis, and 6 cases with mixed tuberculum liver cirrhosis. Seventeen cases had serum alpha-fetoprotein levels of greater than 20 μg/L (upper normal level), which accounts for 73.9%. There were 10 cases in which the hepatoma were located in the lobus sinister, whereas 10 cases had hepatomas in the right lobe and 5 in the middle lobe. According to the Edmondson grading standard, 1 case was grade II, 21 cases were grade III and 1 case was grade IV; 9 cases had a tumor diameter of less than 5 cm, whereas 14 cases had a diameter greater than 5 cm. Four cases were amicula-integrated patients, and the other 19 patients were amicula-incomplete cases. All patients had PVTT that was visible to the naked eye. The 23 pairs of samples of tumor tissue, the corresponding adjacent tissue and the PVTT tissue were all stained by immunohistochemical staining.

#### Patients with HCC without PVTT

A total of 17 cases originated from the resected sample of HCC of active hepatitis without PVTT in the Eastern Hepatobiliary Surgery Hospital from May 2007 to May 2008 (at the same period as the PVTT group). Of all of the cases, 11 were male and 6 were female, and the ages ranged from 31 to 67 years, with an average age of 48. The detection of hepatitis B DNA in all patients was greater than 10^4 ^(10^4^-10^7^) copies/ml. Among the cases, 12 (70.6%) were HbsAg (+), HbeAg (+) and HbcAg (+), whereas 5 (29.4%) were HbsAg (+), HbeAb (+) and HbcAg (+). All the cases were cirrhosis-infected. There were 5 cases of complicating lesser tubercle hepatic cirrhosis, 7 cases of tuberculum majus liver cirrhosis, and 5 cases of mixed tuberculum liver cirrhosis. There were 13 cases (76.5%) in which serum alpha-fetoprotein levels were greater than 20 μg/L (upper normal level). Eleven cases of hepatoma were located in the lobus sinister, whereas 11 cases were located in the right lobe of the liver and 3 cases in the middle lobe of the liver. According to the Edmondson grading standard, two cases were grade II and 15 cases were grade III; there were 3 cases whose tumor diameter was less than 5 cm and 14 cases greater than 5 cm. Five cases were amicula-integrated, and the other 12 cases were amicula-uncompleted. All patients were free from PVTT. The 17 pairs of samples of tumor tissue and the corresponding adjacent tissue were all stained by immunohistochemical staining.

#### Reagents and antibodies

The monoclonal antibody for CXCR4 was purchased from R&D Co. Ltd. The SP (streptavidin-peroxidase) kit was the product of the Zymed Co. Ltd. and was purchased from Beijing Zhongshan Biotechnology Co. Ltd. The following primary antibodies were used: mouse anti-human IgG (R&D) and HRP-conjugated goat anti-mouse secondary antibody (Zymed). All of the other common chemical reagents were purchased from Sigma.

#### Immunohistochemical assay

Streptavidin-peroxidase methods were used. Tissue slices were dewaxed and then washed out. The sections were washed three times with PBS for 5 min. After treatment with 3% H_2_O_2 _solution for 10 minutes, the sections were incubated overnight with anti-CXCR4 antibody (1:100, R&D Co. Ltd) at 4°C. The sections were then washed in PBS and incubated for 1 hour with HRP-conjugated goat anti-mouse secondary antibodies (1:5000, Zymed Co. Ltd). Coloration was achieved through staining with DAB for 3 min. After a series of water-poaching procedures, hematoxylin counterstaining and neutral gum mounting, fluorescent signals were examined using an LSM 5 PASCA1 laser-scanning confocal microscope.

#### Evaluating standard

The slices were examined in a double-blind manner by two different pathologists, and the scores were supplied by the proportion of positive tumor cells and the intensity of the coloring. The standards were defined as follows using the ratio of masculine tumor cells: 0 points represented less than 5%, 1 point represented 5% to 25%, 3 points represented 50% to 75% and 4 points represented greater than 75%. However, the groups could also be classified into the following 4 groups by the intensity of the coloring: 0 represented no coloring, 1 represented stramineous, 2 represented yellow and 3 represented buffy. The products of double multiplication indicated the extent of the cancer. Scores equal to 0 indicate negative (-), whereas scores exceeding 1 indicate positive and scores from 1 to 3 indicate weakly positive (+), 4 to 7 indicate positive (++), and 8 to 12 indicate hadro-positive (+++).

#### Primary hepatoma cells from PVTT

Primary hepatoma cells were prepared from specimens of fresh HCC and PVTT obtained from surgery. The specimens were submerged into RPMI-1640 nutrient solution with antibiotic and then sent to the laboratory at 4°C, followed by the aseptic processing and rejection of blood vessels, amicula, dirty blood and necrotic tissue. The specimens were then cut to 1.0 mm^3 ^and thoroughly washed in D-Hank's solution [11]. The tissues were sheared into starch paste by asepsis scissors. Collagenase solution was added and allowed to digest for 15 to 30 min in a vibrating homeothermia bath, followed by filtration through a cyto-screen (d = 72 μm) and the removal of undigested tissue. Cells were inoculated into plastic Petri dishes; RPMI-1640 was added to the mixture in 5% CO_2_, perfused at 37°C, and then transferred to a 35-mm dish until the cells occupied 80% of the plate.

#### RNAi constructs and gene silencing of CXCR4

A CXCR4-targeting short-hairpin RNA (shRNA) sequence, together with a miRNA-30 loop, was inserted into pGCSIL-GFP vector via AgeI and EcoRI sites. CXCR4-shRNA sequences were designed to target human CXCR4 mRNA (NM_001008540.1). The corresponding virus vector shRNA target was as follows: the sense sequence of the target, from 5' to 3', was CCGGAAGATGATGGAGTAGATGGTGTTCAAGAGAC ACCATCTACTCCATCATCTTTTTTTG; the antisense sequence, from 3' to 5', was ATTCAAAA AAAGATGATGGAGTAGATGGTGTCTCTTGAACACCATCTCTCCATCATCT. The negative control was a hairpin sequence targeting the firefly luciferase gene inserted into the same plasmid at the same sites (Genechem Co. Ltd., Shanghai). The pEGFP-N1-3FLAG vector was used for the construction of an overexpression system for CXCR4 [8]. XhoI and KpnI were the inserting sites. The sequence of CXCR4-XhoI-F was CCGCTCGAGATGTCCATTCCTTTGCCTC from 5' to 3'. The sequence of CXCR4-KpnI-R was CGGGGTACCGTGCTGGAGTGAAAACTTGAAG. These two sequences were used to determine the objective gene by PCR methods [7]. The CXCR4 gene, as amplified by PCR, was completely in accord with sequencing results.

#### Lentivirus infection and migration assay

Primary cells were plated in six-well plates (5 × 10^4 ^cells/well) until cell fusion reached 60%. Then, according to the MOI value (number of lentiviruses per number of cells), appropriate volumes of lentivirus were added to the cells. After 24 h of infection at 37°C, the medium was replaced by fresh medium and incubated for a further 48 h.

The recombinant lentivirus bearing siRNA targeting CXCR4 and the negative control lentivirus were transferred. For the cell migration assay, 1 × 10^4 ^cells from different groups were seeded on a fibronectin-coated polycarbonate membrane insert (6.5 mm in diameter with 8.0-μm pores) in a transwell apparatus and cultured in RPMI-1640. FBS was added to the lower chamber. After incubation for 14 h, the cells on the top surface of the insert were removed by wiping with a cotton swab. Cells that migrated to the bottom surface of the insert were fixed with methanol and stained by Giemsa and then subjected to microscopic inspection.

### Statistical Analysis

Student's *t*-test and ANOVA were used to compare differences in the measurement data among different groups. The chi-squared test was used to compare differences in the rates and proportions between different groups. Regarding the difference comparison of ranked data, the Mann-Whitney nonparametric statistical method was used; P < 0.05 was considered significant, and SPSS 10.0 was used for all analyses. Data are presented as the means ± SD or n/%.

## Results

### CXCR4 expression in tumor tissue and adjacent liver tissue of HCC with PVTT

**Figure 1:**
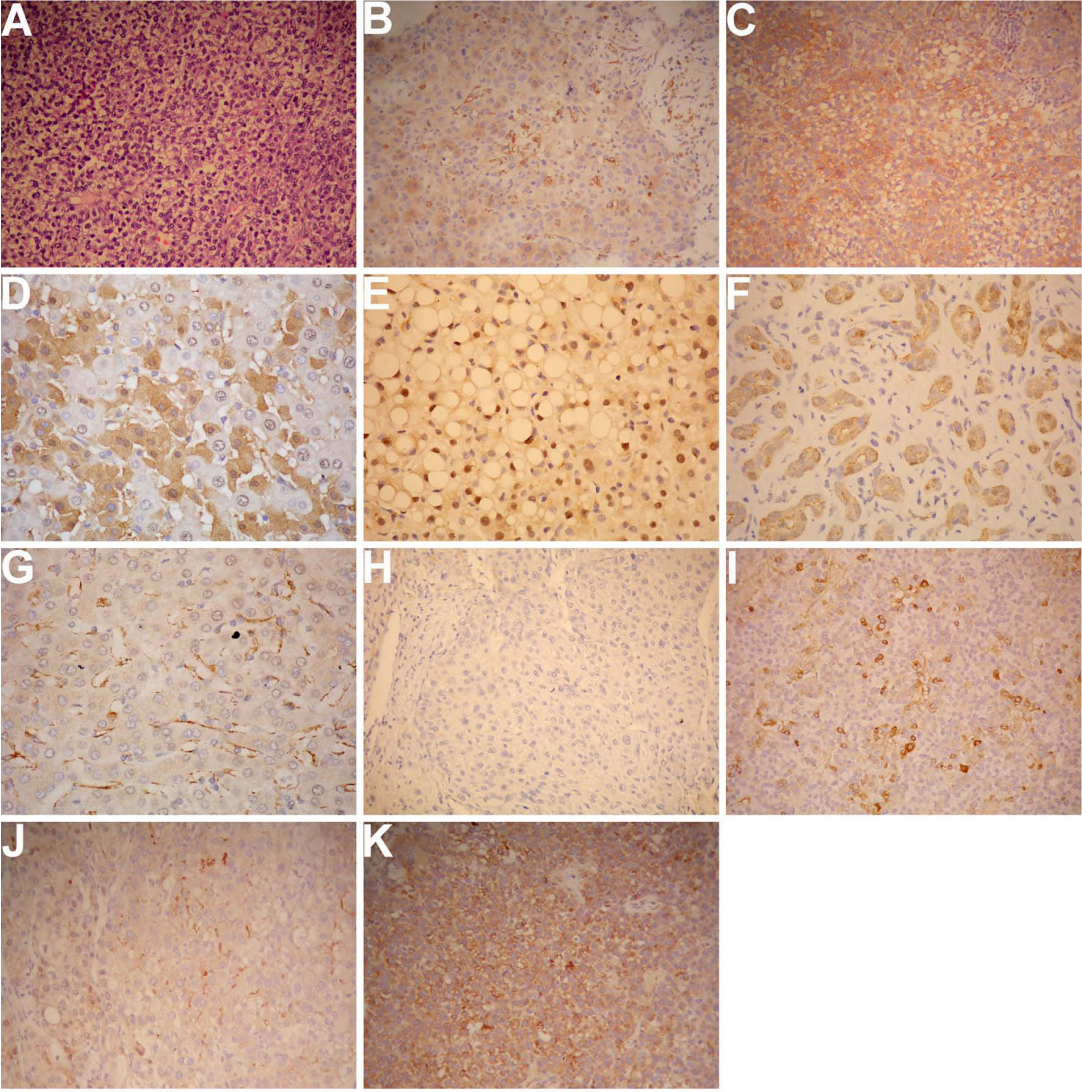
**The expression of CXCR4 in tumor tissue and adjacent liver tissue reflects the characteristic pathology of cancer**. (A-C) Representative images of CXCR4 staining. Tumor tissue was treated with the CXCR4 antibody. The red cells are represented as CXCR4-positive cells. (A) Negative CXCR4-staining cells; (B) Weakly positive staining cells; (C) Positive staining cells. Statistical analysis indicated that 73.9% of all 23 cases were negative, and 6 cases, which occupied 26.1% of all cases, were positive. Magnification: ×200. (D) Representative images of CXCR4 staining. Adjacent liver tissue was treated with the CXCR4 antibody. The red cells indicate CXCR4-positive cells. The CXCR4 cells expressed in inflamed hepatic tissue were mainly located in the cell membrane and cytoplasm. Magnification: 400×. (E-G) CXCR4-positive cells expressed in inflamed hepatic tissue. (E) CXCR4-positive cells located in the liver nucleus; (F) CXCR4-positive cells located in bile canaliculi endothelial cells; (G) CXCR4-positive cells located in hepatic sinusoid endothelial tissue. Magnification: ×400. (H) Negative CXCR4 staining in HCC tissue without PVTT. (I) Positive CXCR4 staining in HCC tissue without PVTT. (J-K) The percentage of positive CXCR4-cells expressed in PVTT tissue is 52.2%. In Figure J, CXCR4 was stained as weakly positive, as opposed to Figure K, which showed positive staining. Magnification: ×200.

Of the 23 specimens of HCC tissue that were stained by immunohistochemistry, 17 (73.9%) exhibited negative staining (Figure 1A). Six samples were positive (Figure 1B and 1C), and the positive ratio was 26.1%. In these samples, 4 were stained as weakly positive, 2 were masculine positive, and CXCR4 was located mainly in the membrane and cytoplasm of hepatoma cells.

The results in the 23 specimens of adjacent liver tissues were quite different. Three cases displayed negative staining after CXCR4 immunohistochemistry, 20 samples were positive, and the ratio of positive staining was 86.0%. The expression of CXCR4 was also mainly detected in the cell membrane and cytoplasm of inflamed hepatic tissue (Figure 1D). As was also expressed in the nucleus (Figure 1E), part of the bile canaliculi endothelial cells and hepatic sinusoid endothelial tissue (Figure 1F and 1G), as well as positive CXCR4, were also observed.

The results of Hematoxylin & Eosin (HE) staining on adjacent liver tissue indicated that the liver was inflamed. The scores were derived from by a proportion of CXCR4-positive cells and coloring intensity to HCC and adjacent liver specimens. The results indicate that the expression levels of CXCR4 in HCC tissue and adjacent liver cells were quite different. We demonstrated that the expression of CXCR4 in adjacent inflammatory liver tissue was dramatically higher than that in tumor tissue (Table 1 P < 0.05).

**Table 1:** Differences in CXCR4 expression in adjacent liver tissue and tumor tissue of HCC with PVTT.

Type of tissue	Number of cases	CXCR4 expression				P value
			
		Negative (-)	Weakly positive (+)	Positive (++)	Hadro-positive (+++)	
Adjacent liver tissue	23	3	6	10	4	0.000^Δ^
Tumor tissue	23	17	4	2	0	

^Δ^Mann-Whitney test

### CXCR4 expression in tumor tissue and adjacent liver tissue of HCC without PVTT

In all 17 specimens of HCC tissue that were stained by immunohistochemistry, 10 cases (58.8%) exhibited negative staining (Figure 1H). Seven samples were positive (Figure 1I), and the positive ratio was 41.2%. In these samples, three cases were stained as weakly positive for CXCR4, and four cases were masculine positive (23.5%).

In the 17 specimens of adjacent liver tissues, four cases (23.5%) displayed negative immunohistochemistry staining for CXCR4, 13 samples were positive, and the ratio of positive staining was 76.5%.

The results of HE staining on the adjacent liver tissue indicated that the liver was inflamed. The scores were determined by a proportion of CXCR4-positive staining cells and coloring intensity to HCC and adjacent liver specimens. The results indicate that the expression levels of CXCR4 in HCC tissue and adjacent liver cells were quite different. Specifically, we found that the expression of CXCR4 in HCC tissue was dramatically lower than that in adjacent liver tissue (Table 2 P < 0.05).

**Table 2:** Differences in CXCR4 expression in adjacent liver tissue and tumor tissue of HCC without PVTT.

Type of tissue	Number of cases	CXCR4 expression				P value
			
		Negative (-)	Weakly positive (+)	Positive (++)	Hadro-positive (+++)	
Adjacent liver tissue	17	4	5	7	1	0.044^Δ^
Tumor tissue	17	10	3	4	0	

^Δ^Mann-Whitney test

### CXCR4 expression in PVTT

In all 23 samples of PVTT tissue, 11 cases exhibited negative immunohistochemistry staining for CXCR4, 12 samples were positive (Figure 1J and 1K), and the positive ratio was 52.2%. The total number of weakly positive and positive samples of CXCR4 expression samples was five, and another two samples exhibited strongly positive staining. Our results indicated that the expression of CXCR4 was mainly located in the membrane and cytoplasm of tumor thrombus cells, which is consistent with a previous report [3].

The positive cell ratio of CXCR4, the staining color intensity of HCC, and tumor thrombus samples were then scored. Previous reports demonstrated that the expression levels of CXCR4 in different HCC tissues and tumor thrombus tissues were quite different [12,13]. We confirmed that the expressions of CXCR4 in tumor thrombus tissues was higher than in HCC tissues (Table 3 p < 0.05).

**Table 3:** Differences in CXCR4 expression in tumor thrombus tissue and tumor tissue.

Type of tissue	Number of cases	CXCR4 expression				P value
			
		Negative (-)	Weakly positive (+)	Positive (++)	Hadro-positive (+++)	
Adjacent liver tissue	23	11	5	5	2	0.044^Δ^
Tumor tissue	23	17	4	2	0	

^Δ^Mann-Whitney test

### CXCR4 expression of PVTT and clinicopathological characteristics of HCC

There was no association between CXCR4 expression of PVTT and the following clinicopathological characteristics of HCC (Additional file 1: Table S1): age of patient, sex of patient, Edmondson grading standard, tumor location, tumor capsule, and liver function (P < 0.05). However, CXCR4 expression was observed to be related to tumor diameter (P > 0.05).

### CXCR4 RNAi in primary hepatoma cells

First, we made a double-stranded DNA oligo with the enzyme-cohesive end in the amphi side, which was directly connected with the RNAi vector after digestion. The construct was then transferred into competent bacterial cells and the positive clones were identified by PCR. After sequencing, the positive clones were proven to be successfully constructed for the lentivirus-vector for RNA interference (RNAi). In this way, we successfully made three shRNA constructs targeting CXCR4 [3,7].

**Figure 2:**
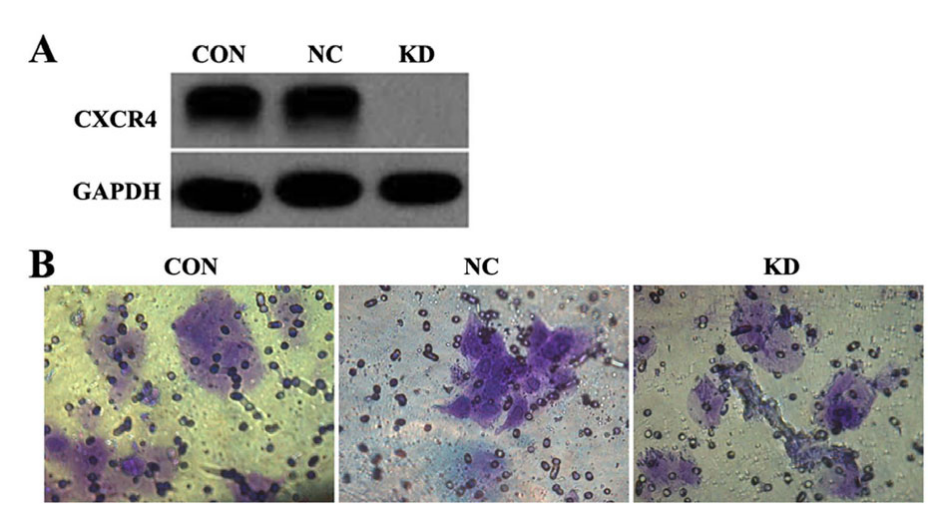
**Knockdown of CXCR4 inhibits the metastasis of PVTT cells *in vitro***. (A) Western blot results indicate the significant knockdown efficiency of CXCR4 expression. (B) In the transwell culture plate, a cell invasion assay was performed. In the negative control, in which cells were infected with the non-silencing lentivirus, the ratio of invasion was quite high, as shown by Giemsa staining. In the knockdown group, as determined by RNAi methods, the ratio of invasion is decreased by CXCR4 silencing (P < 0.05).

We used PCR methods to acquire CXCR4 cDNA and then cloned it into the pEGFP-N1 vector. The products were transformed into competent bacterial cells, and cloning was verified by PCR methods. After sequencing and analyzing the PCR-derived positive clone, the GFP-CXCR4 fusion protein-expressing plasmid was obtained. To test the specificity of the RNAi constructs, we cotransfected into HEK293T cells with the CXCR4 overexpression and CXCR4 RNAi plasmids. After 24 h of incubation, we used western blotting to detect the level of CXCR4 expression (Figure 2A). Significant knockdown of the target was confirmed [14].

### Downregulation of CXCR4 inhibits cell migration

To explore the role of CXCR4 in hepatoma cells, we performed an experiment with invasiveness in transwells in the 24-well culture plate using the cell invasion assay kit. Invasion of the extracellular matrix is an important component of tumor metastasis. The tumor cells can adhere to the vessel wall and extend along the wall. Proteinases such as MMP collagenase could resolve the basilemma of the vessel so that the malignant cells could gain the potential for invasion. The CHEMION cell invasion assay kit provided us with an effective system for the detection of malignant cells crossing the basilemma. We found that the initial inoculating cell numbers were about 20,000. In the internal cell, the culture medium was 300 μL/pore, whereas in the outer compartment, the culture medium was about 500 μL/pore. After culture for 72 h, we employed the MTT assay to find that the average optical density (OD) value in the negative control (infection of negative control of lentivirus cell) was 0.353. After Giemsa staining (Figure 2B), the average OD value became 0.343, which means that the ratio of invasion was 0.971, whereas in the CXCR4-knockdown group the ratio of invasion was 0.747 (P < 0.05). Therefore, we concluded that the knockdown of CXCR4 could inhibit the cell migration of PVTT and that CXCR4 may play a critical role in the metastasis of PVTT.

## Discussion

In this report, we investigated the differential expression of CXCR4 between PVTT and HCC cells. The expression of CXCR4 in tumor thrombus tissue was higher than in HCC tissue, which was consistent with high expression of CXCR4 facilitating the characteristics of metastasis [15,16]. The expression of CXCR4 in HCC tissue was significantly lower than in carcinoma inflammatory liver tissue. As in the data of the group with active hepatitis, the numbers of HBV DNA were all greater than 10^4^. Whether or not the adjacent liver tissue was infected with PVTT, in inflammatory conditions CXCR4 was highly expressed.

In the past several years, the establishment of a series of human HCC cell lines provided an ideal *in vitro *model to study the pathogenesis of liver cancer, metastasis, development and therapy methods in molecular biology. To explore the role of CXCR4 in carcinoma tissue, the primary cells driven from PVTT have been identified. The tumor cells were mostly derived from the primary HCC tissues of patients. Few studies have used PVTT for establishing cell lines; Hu et al. [15] reported that the depletion of 8 bp in a chromosome possibly corresponded with the formation of PVTT when using primary cell culture methods on a PVTT that was primarily focused in the liver, as determined by karyotype analysis and comparative genomic hybridization techniques. Our results confirmed one new HCC cell line derived from human PVTT, which provided sufficient experimental support for the study of the formation mechanism of PVTT. In fact, there were nearly no similar references on the establishment of PVTT cell lines for human hepatoma cancer. Therefore, it is important to study the formation and metastasis mechanisms of PVTT in this primary cell line.

To gain insight into the role of CXCR4 in HCC tumorigenesis and metastasis, we employed lentivirus-mediated shRNA to knock down CXCR4 expression in PVTT cells. After screening the siRNA targets, we found the most significant knockdown targeting the expression of CXCR4. The chemokine receptor CXCR4 is implicated in the metastasis of various cancers. The association of CXCR4 expression with HCC bone metastasis and patient survival was recently reported. CXCR4 expression in primary HCCs may be an independent risk factor for bone metastasis and associated with poor clinical outcome [17]. Our transwell results indicated that depletion of CXCR4 expression resulted in significant inhibition of PVTT cell migration. These data extend the critical role of CXCR4 in promoting the migration of cancer cells. The central role of CXCR4 in cancer metastasis also raises the question of whether CXCR4 can serve as an important diagnostic target in the detection and treatment of cancer. Additionally, it is important to further establish the mechanisms that result in increased CXCR4 expression andpotentially target such pathways in cancer treatment. Thus, understanding the mechanisms that normally regulate CXCR4 expression and function should prove useful in the treatment and prevention of cancer metastasis.

## Conclusions

We determined that the expression of CXCR4 in PVTT tissue was greater than that in liver cancer tissue and that the downregulation of CXCR4 by RNA interference significantly impaired the invasive ability of PVTT cells. It is possible that CXCR4 plays a critical role in the development of PVTT in HCC. The potential siRNA target we screened may have an advantageous curative effect on HCC.

## Competing interests

The authors declare that they have no competing interests.

## Authors' contributions

NL and SQC conceived, coordinated and designed the study and contributed to the acquisition, analysis and interpretation of data and drafted the manuscript. WXG performed the experiments and were involved in drafting the article. JS and JX selected archived samples and participated in the study design and interpretation of the results. HSH participated in sample collection and data acquisition. All authors have read and approved the final manuscript.

## Supplementary Material

Additional file 1 **Table S1: Association between CXCR4 expression of PVTT and clinicopathological characteristics of HCC**. CXCR4 expression of PVTT was observed to be related to tumor diameter. There was no association between CXCR4 expression and the following clinicopathological characteristics of HCC: age, sex, Edmondson grading, tumor location, tumor capsule, and liver function. Click here for file
