# Supplementary material for: Expression of the chemokine receptor CXCR4 in human hepatocellular carcinoma and its role in portal vein tumor thrombus
Source: J Exp Clin Cancer Res. 2010 Nov 27;29(1):156. doi: 10.1186/1756-9966-29-156 (PMC3002328; doi:10.1186/1756-9966-29-156)
Supplement: Additional file 1 — Table S1: Association between CXCR4 expression of PVTT and clinicopathological characteristics of HCC. CXCR4 expression of PVTT was observed to be related to tumor diameter. There was no association between CXCR4 expression and the following clinicopathological characteristics of HCC: age, sex, Edmondson grading, tumor location, tumor capsule, and liver function. [file 1756-9966-29-156-S1.DOC]

**Table S1.**

**Association between CXCR4 expression of PVTT and clinicopathological characteristics of HCC.**

| Clinicopathological characteristics | CXCR4 expression | | P value |
| --- | --- | --- | --- |
|  | Negative (-)  (n=11) | Positive (+ to +++）  (n=12) |  |
| Age span (years) | 39.73±9.57 | 44.17±9.97 | 0.288^▲^ |
| Sex |  |  | 0.156^△^ |
| Male | 5 | 9 |  |
| Female | 6 | 3 |  |
| AFP (μg/L) |  |  | 0.903^△^ |
| ≤20 | 8 | 9 |  |
| >20 | 3 | 3 |  |
| Edmondson Grading |  |  | 0.166^△^ |
| Ⅱ | 1 | 0 |  |
| Ⅲ | 10 | 11 |  |
| Ⅳ | 0 | 1 |  |
| Tumor location |  |  | 0.165^△^ |
| Left hepatic lobe | 5 | 3 |  |
| Right hepatic lobe | 5 | 5 |  |
| Middle hepatic lobe | 1 | 4 |  |
| Tumor diameter (cm) |  |  | 0.024^△^ |
| ≤5 | 7 | 2 |  |
| >5 | 4 | 10 |  |
| Tumor capsule |  |  | 0.242^△^ |
| Integrated | 3 | 1 |  |
| Incomplete | 8 | 11 |  |
| Child classification |  |  | 0.530^△^ |
| A | 3 | 5 |  |
| B | 7 | 6 |  |
| C | 1 | 1 |  |

^▲^Analysis of variance

^△^Non-parametric test: Mann-Whitney test
